# Correction: Social preferences in the public goods game–An Agent-Based simulation with EconSim

**DOI:** 10.1371/journal.pone.0317061

**Published:** 2024-12-31

**Authors:** Christoph Bühren, Jan Haarde, Christian Hirschmann, Janis Kesten-Kühne

The first author, Christoph Bühren, is incorrectly noted as the corresponding author. The correct corresponding author is Christian Hirschmann. Christian Hirschmann’s email address is: christian.hirschmann@tu-clausthal.de.
