# Supplementary figures and images for: Social preferences in the public goods game–An Agent-Based simulation with EconSim
Source: PLoS One. 2023 Mar 15;18(3):e0282112. doi: 10.1371/journal.pone.0282112 (PMC10016715; doi:10.1371/journal.pone.0282112)

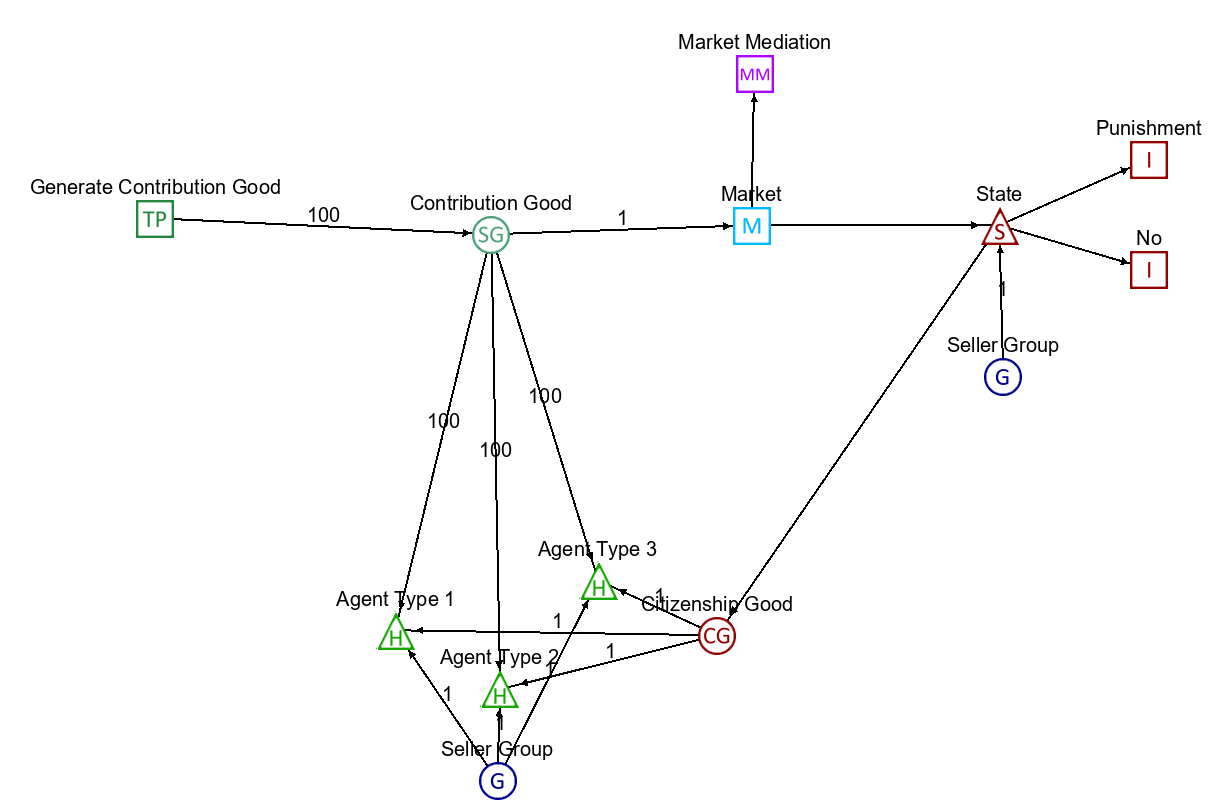

Supplement: S1 Fig — (TIF) [file pone.0282112.s001.tif]

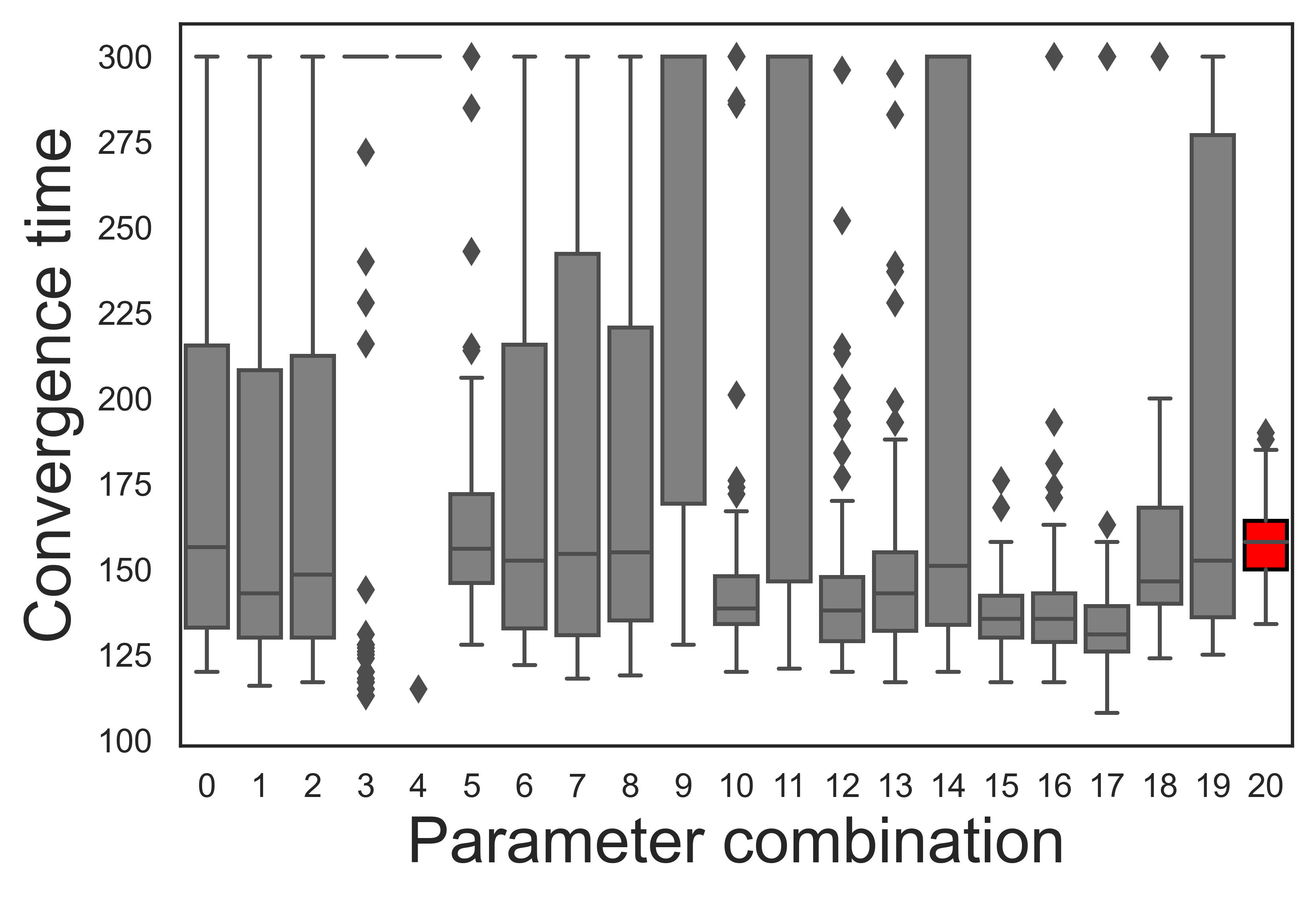

Supplement: S2 Fig — In red: chosen parameter configuration in sections 3.6 and (slightly adjusted) 3.7. (TIF) [file pone.0282112.s002.tif]

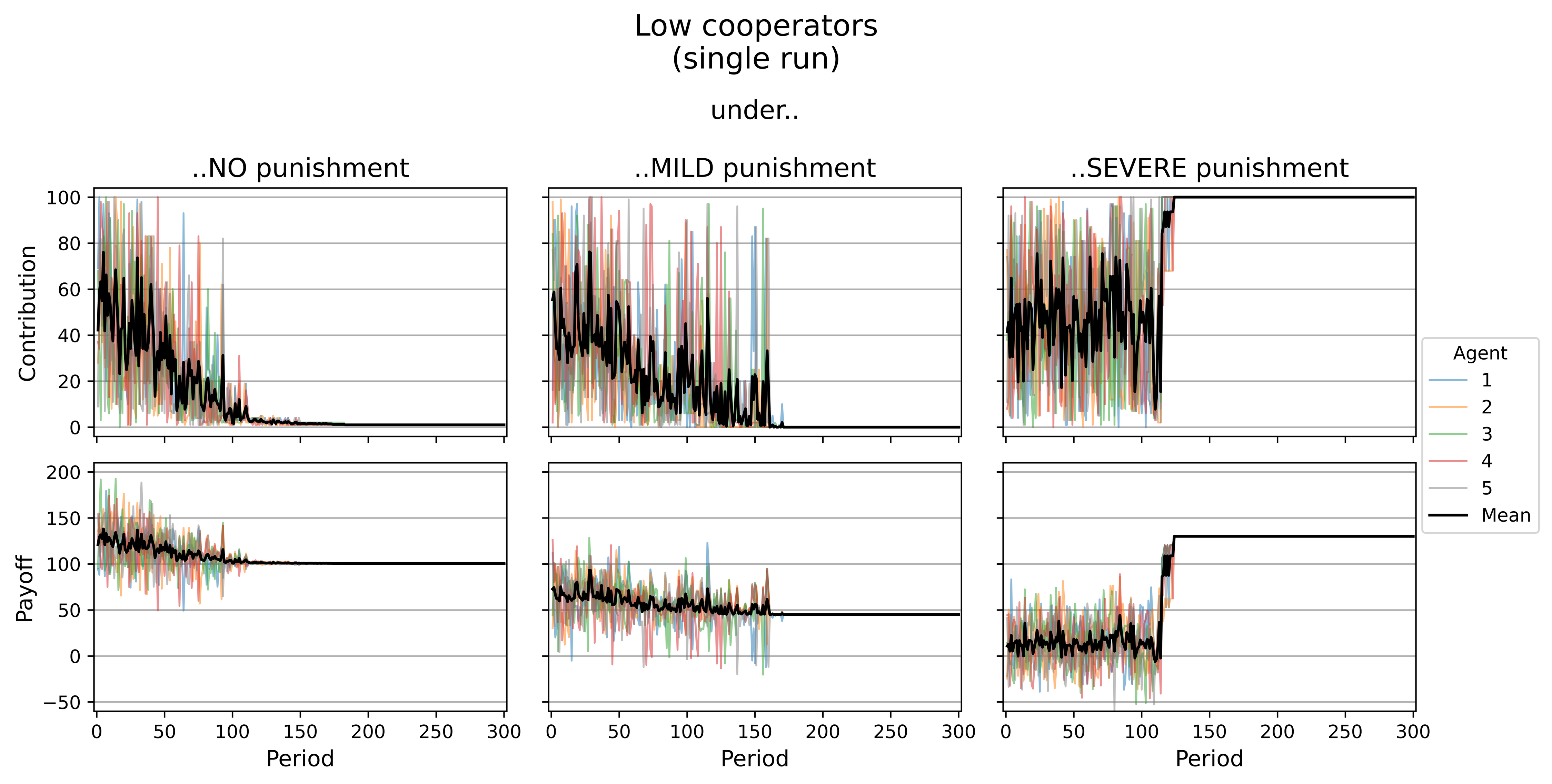

Supplement: S3 Fig — (TIF) [file pone.0282112.s003.tif]

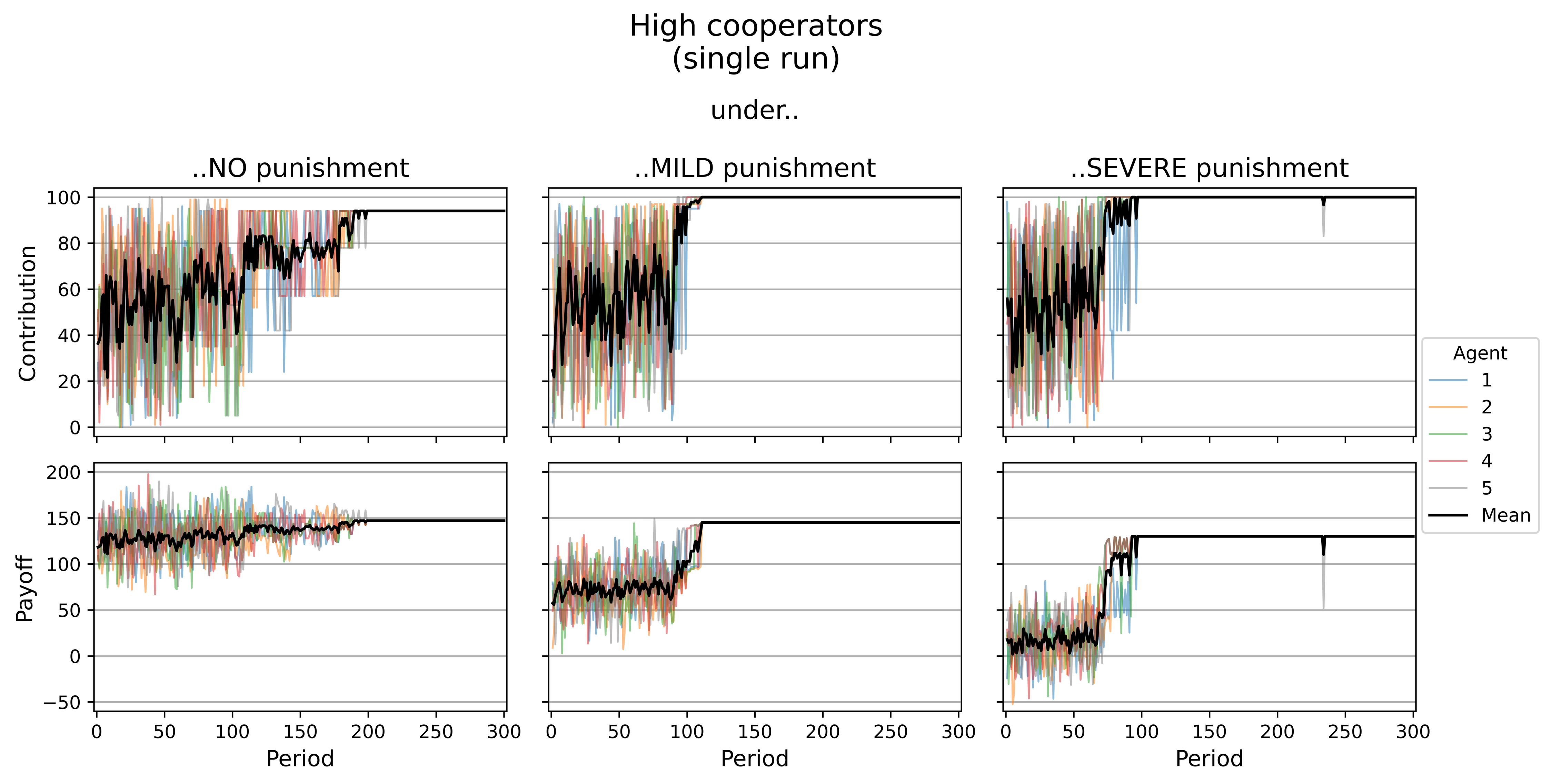

Supplement: S4 Fig — (TIF) [file pone.0282112.s004.tif]

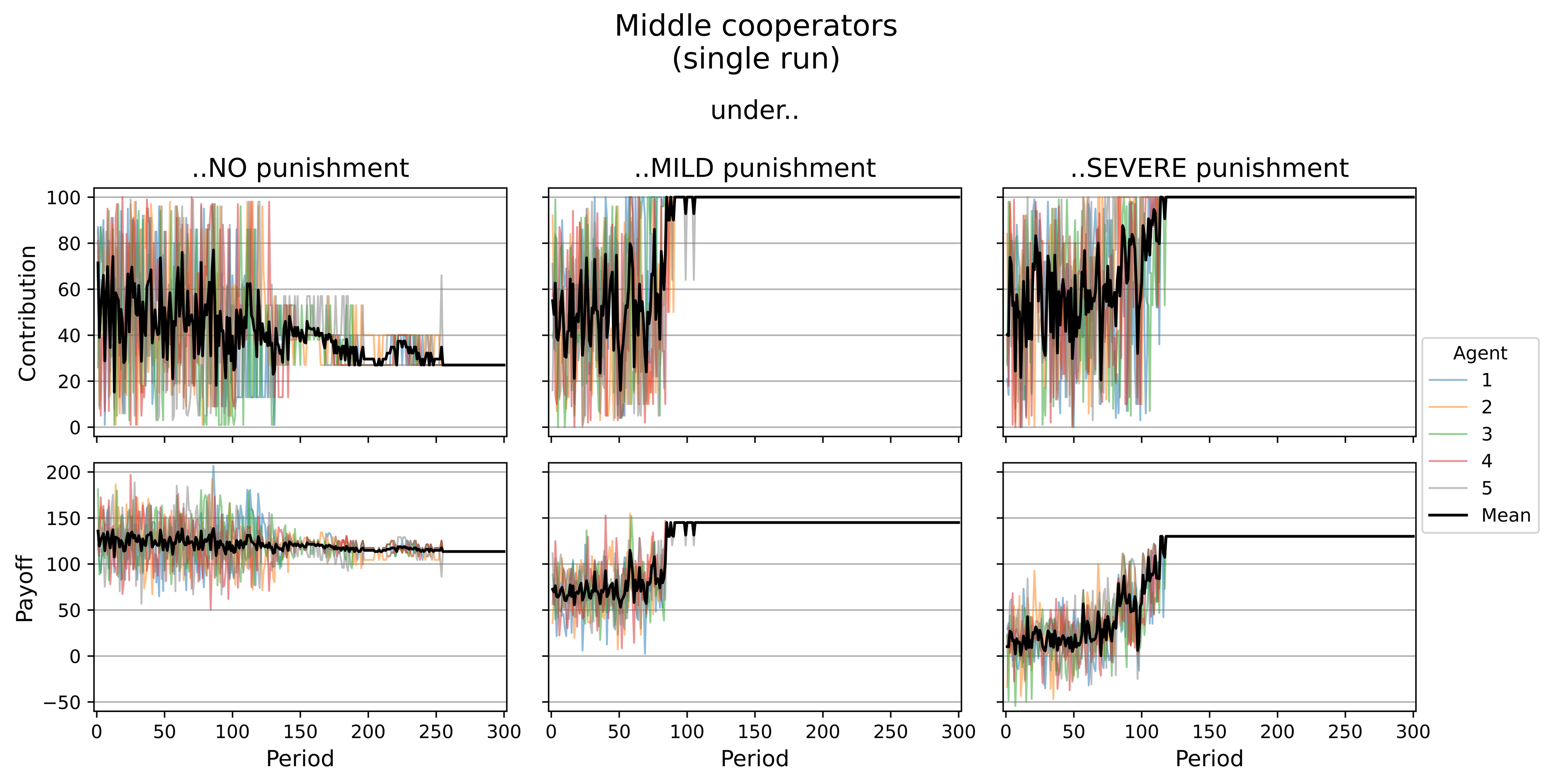

Supplement: S5 Fig — (TIF) [file pone.0282112.s005.tif]

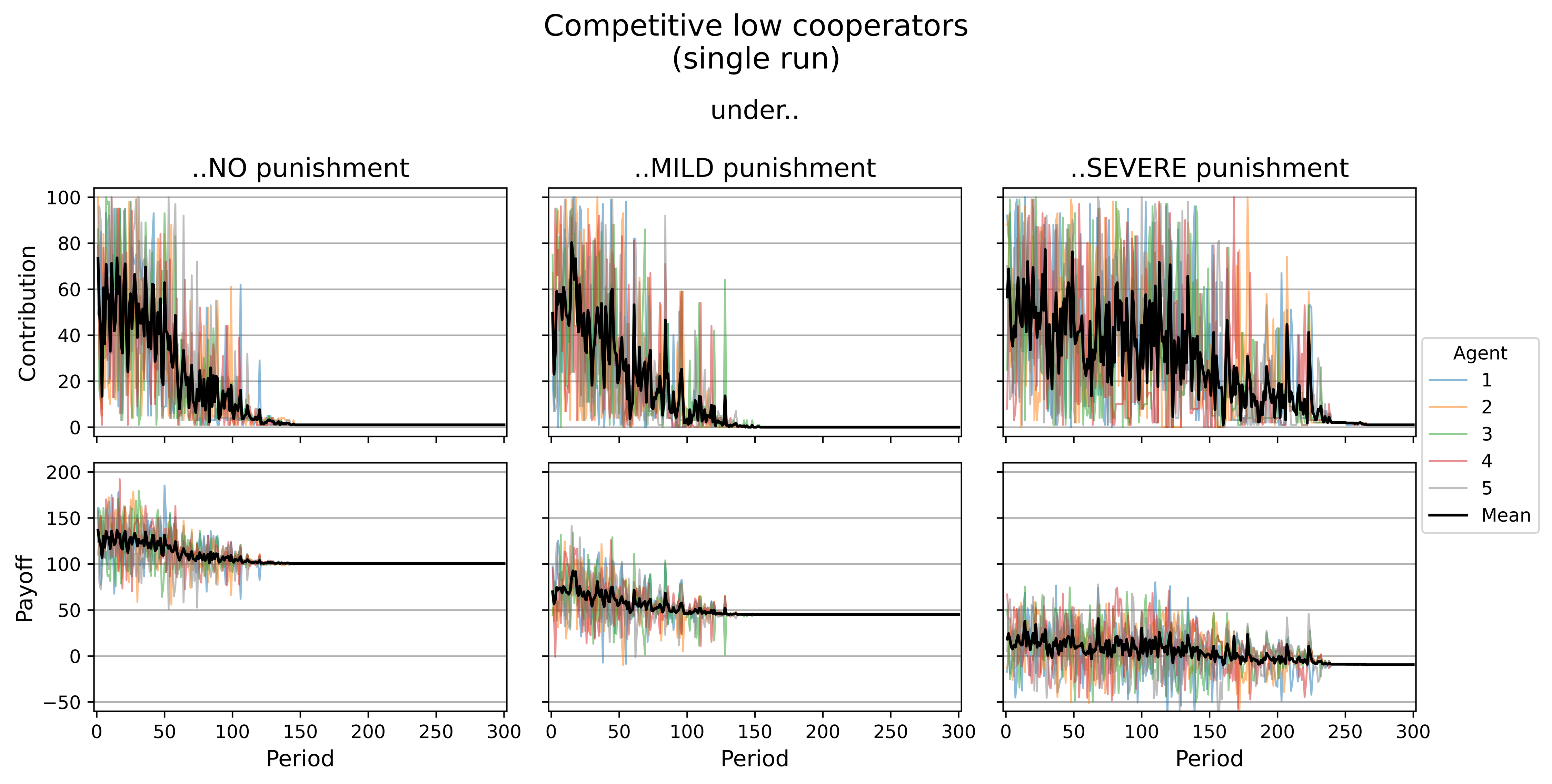

Supplement: S6 Fig — (TIF) [file pone.0282112.s006.tif]
