# Supplementary material for: Social preferences in the public goods game–An Agent-Based simulation with EconSim
Source: PLoS One. 2023 Mar 15;18(3):e0282112. doi: 10.1371/journal.pone.0282112 (PMC10016715; doi:10.1371/journal.pone.0282112)
Supplement: S1 Table — Underlined: changes relative to Combination 0. (PDF) [file pone.0282112.s007.pdf]

S1 Table: Sensitivity analysis of the reinforcement learning parameters. Underlined: changes relative to Combination 0.

**Number**	**0**	**1**	**2**	**3**	**4**	**5**	**6**	**7**	**8**	**9**	**10**
Epsilon greedy	0.02	0.01	0.05	0.02	0.02	0.02	0	0.02	0.02	0.02	0.02
Initial attractivity	67	67	67	67	67	67	67	50	85	67	67
Exponential smoothing (new/old)	0.75/0.25	0.75/0.25	0.75/0.25	0.5/0.5	0.25/0.75	1/0	0.75/0.25	0.75/0.25	0.75/0.25	0.75/0.25	0.75/0.25
Initial temperature	1	1	1	1	1	1	1	1	1	1	1
Temp. variation	0.05	0.05	0.05	0.05	0.05	0.05	0.05	0.05	0.05	0.05	0.05
Lower/upper bound temperature	[0.01/1]	[0.01/1]	[0.01/1]	[0.01/1]	[0.01/1]	[0.01/1]	[0.01/1]	[0.01/1]	[0.01/1]	[0.01/1]	[0.01/1]
Attractivity cooldown	0.01	0.01	0.01	0.01	0.01	0.01	0.01	0.01	0.01	0.03	0.005
Imitation	0.05	0.05	0.05	0.05	0.05	0.05	0.05	0.05	0.05	0.05	0.05
Imitation of	Any agent	Any agent	Any agent	Any agent	Any agent	Any agent	Any agent	Any agent	Any agent	Any agent	Any agent
**Results**											
Avg. speed of adaption in rounds	288.66	279.30	282.49	300.00	300.00	246.85	282.76	277.38	285.22	295.60	270.73
Standard deviation of adaption speed	35.81	47.30	38.99	0.00	0.00	63.44	41.24	46.38	37.40	24.97	49.68
Percentage full convergence	13.00	20.00	21.00	0.00	0.00	46.00	20.00	25.00	18.00	4.00	34.00
Average contribution	1.83	1.54	1.44	4.35	10.62	1.00	1.74	1.61	1.74	4.42	0.88
Standard deviation of average contribution	1.40	1.28	1.32	2.02	5.14	1.36	1.55	1.52	1.38	2.91	0.85

to be continued

S1 Table continued: Sensitivity analysis of the reinforcement learning parameters. Underlined: changes relative to Combination 0.

**Number**	**0**	**11**	**12**	**13**	**14**	**15**	**16**	**17**	**18**	**19**	**20**
Epsilon greedy	0.02	0.02	0.02	0.02	0.02	0.02	0.02	0.02	0.1	0	0.01
Initial attractivity	67	67	67	67	67	67	67	67	67	67	50
Exponential smoothing (new/old)	0.75/0.25	0.75/0.25	0.75/0.25	0.75/0.25	0.75/0.25	0.75/0.25	0.75/0.25	0.75/0.25	0.75/0.25	0.75/0.25	1/0
Initial temperature	1	1	1	1	1	1	1	1	1	1	1
Temp. variation	0.05	0.05	0.05	0.05	0.05	0.05	0.05	0.05	0.05	0.05	0.05
Lower/upper bound temperature	[0.01/1]	[0.01/1]	[0.01/1]	[0.01/1]	[0.01/1]	[0.01/1]	[0.01/1]	[0.01/1]	[0.01/1]	[0.01/1]	[0.01/1]
Attractivity cooldown	0.01	0.01	0.01	0.01	0.01	0.01	0.01	0.01	0.03	0.005	0.005
Imitation	0.05	0.02	0.1	0.05	0.02	0.1	0.2	0.5	0.2	0.02	0.2
Imitation of	Any agent	Any agent	Any agent	Best agent	Best agent	Best agent	Any agent	Any agent	Any agent	Any agent	Any agent
**Results**											
Avg. speed of adaption in rounds	288.66	300.00	256.53	244.73	294.59	195.18	233.67	259.27	277.31	289.54	217.69
Standard deviation of adaption speed	35.81	0.00	64.19	68.78	28.27	73.13	79.87	72.97	54.49	33.11	69.44
Percentage full convergence	13.00	0.00	35.00	46.00	5.00	70.00	42.00	24.00	15.00	11.00	59.00
Average contribution	1.83	3.33	1.11	0.64	1.65	0.42	1.21	2.01	3.26	1.64	0.69
Standard deviation of average contribution	1.40	1.87	1.15	0.78	1.20	0.78	1.53	1.84	3.08	1.12	1.03
