# Supplementary material for: Social preferences in the public goods game–An Agent-Based simulation with EconSim
Source: PLoS One. 2023 Mar 15;18(3):e0282112. doi: 10.1371/journal.pone.0282112 (PMC10016715; doi:10.1371/journal.pone.0282112)
Supplement: S1 File — (PDF) [file pone.0282112.s008.pdf]

## Pseudocode NESASS

**Input:** $a_t$ (chosen decision), $a_{t-1}^*$ (best decision in last period if unique), $q(t-1)$ (attractivity vector), $\alpha^{new}$ and $\alpha^{old}$ (weighting factors combining new reward with old attractivity), $r(s_t, a_t)$ (reward for chosen decision in current state), $\mu$ (rationality parameter), $\mu_\Delta$ (variation of rationality parameter), $\mu_{init}$ (initial value of $\mu$), $\varphi$ (depreciation parameter), $\underline{\mu}$ (lower bound for $\mu$), $\overline{\mu}$ (upper bound for $\mu$)

*First, all attractivity values are updated*

**for all** $a \in \mathrm{A}$ **do**

*Chosen action: combine received reward and old attractivity value to new attractivity value*

**if** $a = a_t$ **then** $q(a,t) \coloneqq \alpha^{new} \cdot r(s_t, a) + \alpha^{old} \cdot q(a, t-1)$

*All other actions: depreciate old attractivity value*

**else** $q(a,t) \coloneqq (1-\varphi) \cdot q(a, t-1)$

$q^{max} \coloneqq \max_{a \in A} q(a,t)$ *Get highest attractivity value; needed for normalization*

$nBestDec \coloneqq 0, a_t^* \coloneqq null, \mu^{new} \coloneqq 0$

**for all** $a \in A$ **do**

**if** $q^{max} = q(a,t)$ **then**

$nBestDec := nBestDec + 1,\ a_t^* \coloneqq a$ *Count actions with the highest attractivity value*

*If there is only one best decision and…*

**if** $nBestDec = 1$ and $a_{t-1}^* \neq null$ **then**

*… the best decision has not changed over the last iteration…*

**if** $a_{t-1}^* = a_t^*$ **then**

*… then decrease the value of $\mu$*

$\mu^{new} \coloneqq max\left(\underline{\mu}, \mu \cdot (1-\mu_\Delta)\right)$

*… the best decision has changed over the last iteration…*

**else**

*… increase the value of $\mu$*

$\mu^{new} \coloneqq min\left(\mu(t-1) \cdot (1+\mu_\Delta), \overline{\mu}\right)$

*Otherwise, if there is not only one best decision and/or no last best decision, do not vary $\mu$*

**else**

$\mu^{new} \coloneqq \mu$

$p(t) \coloneqq zeros[n], cumprob \coloneqq zeros[n]$

*Calculate the probability of every action function based on attractivity values and* $\mu$ *with a softmax function*

**for all** $a \in A$ **do**

$$p(a,t) := \frac{\exp\left(\frac{q(a,t)}{q^{max} \cdot \mu}\right)}{\sum_{\tilde{a} \in A} \exp\left(\frac{q(\tilde{a},t)}{q^{max} \cdot \mu}\right)}$$

*Calculate the cumulated probability; needed for drawing an action*

**for all** $a \in A$ **do**

$$cumprob[a] \coloneqq \sum_{\tilde{a} \in \{A | \tilde{a} \leq a\}} p(\tilde{a}, t)$$

**return** $cumprob, q(t), \mu^{new}, a_t^*$

**Additional comments:**

The hierarchy of our agents' decision-making is as follows:

1. With a given probability the agent chooses another agent's decision from the last round
2. The agent chooses with $\epsilon \cdot (1 - \varphi)$ probability an action by drawing from the uniform distribution over the strategy space
3. The agent draws its decision from the cumulative probability distribution $cumprob$

However, the decision (chosen from one of these three possibilities) is used for learning.
